# Barriers and Facilitators of the Uptake of Human Immunodeficiency Virus (HIV) Services Among Transgender Women: A Mixed-Methods Study in Delhi, India

**DOI:** 10.7759/cureus.110798

**Published:** 2026-06-13

**Authors:** Somdatta Patra, Sanjiv Kumar Bhasin, Naudibya Majhi, Praveen Kumar, J. K. Mishra

**Affiliations:** 1 Department of Community Medicine, University College of Medical Sciences, Delhi, IND; 2 Department of Community Medicine, Employee's State Insurance Corporation (ESIC) Medical College, Noida, IND; 3 Department of Public Health, Delhi State AIDS Control Society, Delhi, IND; 4 Division of Targeted Intervention, Delhi State AIDS Control Society, Delhi, IND

**Keywords:** aids, discrimination, health system, hiv, stigma, transgender

## Abstract

Background: Transgender women face higher human immunodeficiency virus (HIV) risk due to stigma, social marginalization, and high-risk behavior.

Aim: This study aimed to explore the barriers and facilitators to HIV service uptake among transgender women to improve healthcare access.

Methodology: A mixed-methods study was conducted among transgender women in Delhi, India. Data were collected from March to August 2023, using both quantitative and qualitative approaches. The quantitative study involved 170 participants from targeted intervention (TI) sites, focusing on sociodemographic characteristics, health profiles, and service utilization. The qualitative part included four focus group discussions (FGDs) and five in-depth interviews (IDIs) with transgender women, community leaders of the transgender community, and healthcare providers (medical officer, laboratory technician, and counsellor). Data analysis included statistical measures for quantitative data and thematic analysis for qualitative data to identify factors affecting service utilization. Ethical approval was obtained.

Results: Among 170 transgender women, 48.2% were aged 20-30 years, and 43.5% lived within the transgender community. Nearly one-fifth (19.4%) reported a chronic health condition, while 26.5% had visited a health facility in the previous 30 days. HIV testing uptake was high, with 98.2% reporting periodic testing at TI sites and 12.5% testing HIV positive. Qualitative findings revealed multiple barriers to HIV service utilization, including internalized stigma, fear of disclosure, discriminatory experiences in healthcare settings, housing and financial insecurity, difficulties in condom negotiation, and limited transgender-sensitive services. Peer support, non-governmental organization (NGO) linkage, and transgender-inclusive TI services facilitated access.

Conclusion: Stigma, discrimination, and lack of a transgender-sensitive health system significantly hinder HIV prevention and antiretroviral therapy (ART) access for transgender individuals. Fear of disclosure, healthcare system deficiencies, and financial instability further complicate access. The study highlights the need for stigma reduction, trans-friendly healthcare, and economic empowerment to improve HIV-related services in this marginalized community.

## Introduction

In India, around 3.1% of transgender individuals are estimated to be living with human immunodeficiency virus (HIV), which is much higher than the 0.22% prevalence in the general population [1]. The transgender community includes individuals whose sex assigned at birth (male or female) does not match the gender they may identify with [2,3]. Transgender women [4], people who were assigned male sex at birth but identify themselves as female, are considered at an increased risk of acquiring HIV and other sexually transmitted infections (STIs). Their increased risk is linked to engaging in high-risk behaviors and being socially marginalized or isolated. Social challenges like violence, lack of gender recognition, and exclusion from regular jobs and the education system make them more vulnerable to infection and disease [5-7]. These struggles oftentimes affect their mental health and also limit their access to essential HIV-related services [8-10]. Despite targeted HIV prevention and treatment services being available under the National AIDS Control Programme [11], evidence on the barriers and facilitators influencing the uptake of these services among transgender women in the Indian context remains limited. Understanding these factors is essential for designing more responsive and transgender-inclusive HIV programs. Therefore, this study was undertaken to explore the barriers and facilitators to the uptake of HIV services among transgender women.

Some part of this study was previously presented as an oral presentation at the 52nd National Conference of the Indian Association of Preventive and Social Medicine on April 12, 2025, at Government Medical College (GMC), Srinagar, Jammu and Kashmir, India.

## Materials and methods

A mixed-methods study was conducted with transgender women aged 18 years and above, registered at targeted intervention (TI) sites in the North East, East, and Shahdara districts of Delhi, India. In Delhi, TI programs for transgender individuals are implemented through 10 TI sites under the National AIDS Control Programme through community-based organizations or non-governmental organizations (NGOs). These sites aim to reduce the risk of HIV and other infections by offering free health services, including periodic HIV testing, counselling, and STI treatment. Outreach workers engage with the community to raise awareness, distribute condoms, and ensure linkage to healthcare facilities.

The sample size and the selection of the study site were based on convenience considering the accessibility and available time for data collection. Data were collected from March to August 2023. The inclusion criteria were the people who (i) identified themselves as transgender women, (ii) were aged 18 years or above, (iii) were registered beneficiaries of the selected TI site, and (iv) visited the TI site during the period of data collection. Exclusion criteria were transgender women from whom written informed consent could not be obtained. The quantitative component involved a community-based cross-sectional study using a semi-structured interview schedule with 170 transgender women who were registered with the selected TI site and were visiting the TI site on the days of data collection. The aim of the quantitative component was to find out the sociodemographic and health profile and health behavior of the study participants. For the collection of data for the quantitative component of the study, a pre-tested, semi-structured, interviewer-administered questionnaire (developed by the authors; see Appendices) was used to record (i) sociodemographic data, (ii) self-reported morbidities including HIV status and addiction history, and (iii) health service utilization. The questionnaire had both open- and closed-ended questions.

The qualitative component included four focus group discussions (FGDs), two with transgender women and two with community leaders (gurus). A total of 29 transgender women participated in the FGDs. Additionally, five in-depth interviews (IDIs) were conducted, including two with people living with HIV/AIDS (PLHIV) and three with healthcare providers, such as a medical officer, a counsellor, and a laboratory technician. Participants for the FGDs and IDIs (transgender women living with HIV) included in the qualitative component were purposively selected from transgender women who had participated in the quantitative component of the study. The three healthcare providers included in the study were employed at the selected TI site.

All interviews (both quantitative and qualitative components of the study) and FGDs were conducted in person after receiving written informed consent from the participants. Each study participant was contacted personally by the investigators at the TI site and at a time suitable to the study participants. After explaining the nature and purpose of the study and assuring confidentiality, a consent form and a participant information sheet were handed over to the participant. Sufficient time was spent to explain the importance of the study, and doubts, if any, were cleared. The participants were requested to provide correct and complete information.

The Institutional Ethics Committee-Human Research (IEC-HR) of University College of Medical Sciences approved this study (approval number: IECHR-2021-57-2-R1; date: November 28, 2022).

Data analysis

Quantitative Component of the Study

The collected data were entered into a computer-based spreadsheet using MS Excel (Microsoft Corporation, Redmond, Washington, United States) and analyzed using IBM SPSS Statistics for Windows, Version 20.0 (IBM Corp., Armonk, New York, United States). Statistical analysis included the calculation of means and proportions. For quantitative variables, mean and standard deviation were computed for normally distributed data, whereas median and interquartile range were reported for data with non-normal distribution. For categorical variables, proportions were calculated.

Qualitative Component of the Study

Audio recording was done for all FGDs and IDIs which were transcribed verbatim. The transcripts were carefully reviewed multiple times to gain an understanding of facilitators and barriers faced by transgender women while accessing HIV-related services. A combination of deductive and inductive approaches was used for data analysis. The deductive approach was guided by the research questions and existing literature, while the inductive approach allowed for the identification of new themes emerging from the data. A coding scheme was developed and progressively refined over time to ensure flexibility in accommodating new themes. Thematic analysis was conducted, and the identified themes were documented. The relevant data sources from which these themes were derived are indicated in parentheses throughout the Results section. Additionally, direct quotations associated with the themes are presented in double quotes within the Results section.

## Results

Demographic profile of the study participants

Among the 170 study participants, 20 (11.76%) were aged below 20 years, 82 (48.23%) were between 20 and 30 years, 35 (20.58%) were between 30 and 40 years, and 33 (19.41%) were aged more than 40 years. Regarding educational status, 43 (25.30%) participants were illiterate, 56 (32.94%) had education up to the eighth standard, 60 (35.29%) had studied between the ninth and 12th standards, and 11 (6.47%) were graduates or had higher education. More than half of the participants, 88 (51.76%), were engaged in singing and dancing activities as their occupation. Sex work was reported by 37 (21.76%) participants, while 24 (14.11%) were involved in begging on the streets. Office work was reported by 15 (8.82%) participants, one (0.58%) worked in a parlor, and five (2.94%) participants were unemployed. The median monthly income of the participants was Indian national rupees (INR) 15,000 with an interquartile range of INR 11,500-20,000. Seventy-four (43.52%) participants were living with the transgender community, 51 (30%) were living alone, 33 (19.41%) were living with their birth family, and 12 (7.05%) were residing with their partner. Most participants were single (117 (68.82%)), while 41 (24.11%) were in a relationship. Married participants were 11 (6.47%), and one (0.58%) participant was divorced. Tobacco use was reported by 66 (38.82%) participants, alcohol use by 28 (16.47%), and "ganja" or other drug use by eight (4.70%) participants. Eighty (47.05%) participants reported no substance use (Table 1).

**Table 1:** Distribution of the sociodemographic characteristics of the study participants (n=170) ^*^Many of the participants were involved in multiple jobs; the occupation mentioned here is the primary source of income at the time of interview ^#^More than one form of substance abuse was present in some of the participants

Variables	Number (%)
Age	
<20 years	20 (11.76)
20-30 years	82 (48.23)
30-40 years	35 (20.58)
>40 years	33 (19.41)
Education	
Illiterate	43 (25.30)
Up to the 8th standard	56 (32.94)
9th to 12th standards	60 (35.29)
Graduate or higher	11 (6.47)
Occupation^*^	
Singing and dancing	88 (51.76)
Sex work	37 (21.76)
Begging on the streets	24 (14.11)
Office work	15 (8.82)
Parlor	1 (0.58)
Unemployed	5 (2.94)
Monthly income (Indian national rupees)	
Median (interquartile range)	15,000 (11,500-20,000)
Living arrangement	
Transgender community	74 (43.52)
Alone	51 (30)
Birth family	33 (19.41)
Partner	12 (7.05)
Relationship status	
Single	117 (68.82)
In a relationship	41 (24.11)
Married	11 (6.47)
Divorced	1 (0.58)
Substance use^#^	
Tobacco	66 (38.82)
Alcohol	28 (16.47)
Ganja and other drugs	8 (4.70)
None	80 (47.05)

Most of them lived without family and social support. Usually, for any work to be done, they had to pay a rate higher than usual. "The room that costs Rs (Indian National Rupee) 500 is available for 1500 for me" (a transgender woman during FGD). Another participant echoed, "While everyone else is staying here for Rs 6,000, I pay the highest rent, Rs 8,000". Another participant described experiencing housing-related discrimination: "After looking at my identity card (as "transgender" was mentioned), the landlord refused to rent the house to me" (FGD participant).

Most of the time, transgender women were disowned by their birth family, which also led them to drop out of school, and they are sheltered by the gurus (community leaders). "Nobody likes to give any job to a TG person, and they start working as sex workers" (counsellor discussing many times a transgender person is forced into sex work).

Morbidity profile (self-reported) and health service utilization

Among the study participants, 33 (19.41%) reported having at least one chronic health problem. The most commonly reported condition was diabetes mellitus, reported by 10 (5.88%) participants, followed by dermatological conditions in eight (4.70%) participants and hypertension in seven (4.11%) participants. Musculoskeletal disorders and hypothyroidism were each reported by four (2.35%) participants, while psychiatric illnesses were reported by two (1.17%) participants (Table 2).

**Table 2:** Morbidity profile (self-reported) and health service utilization by transgender women ^*^The term "Bengali doctor" is commonly used in this part of India to refer to informal healthcare providers or unqualified practitioners ^#^Categories are not mutually exclusive. One participant reported multiple health conditions

Variable	Number (%)/mean±SD
Presence of chronic health problems (self-reported)	33 (19.41)
Reported chronic conditions^#^	
Diabetes mellitus	10 (5.88)
Dermatological conditions	8 (4.70)
Hypertension	7 (4.11)
Musculoskeletal disorders	4 (2.35)
Hypothyroidism	4 (2.35)
Psychiatric illnesses	2 (1.17)
Visited any health facility in the past 30 days	45 (26.47)
Type of health facility visited (n=45)	
Private clinics/"Bengali doctors"^*^	29 (64.44)
Government health facilities	12 (26.66)
Pharmacy	4 (8.89)
Mean±(SD) visits to the targeted intervention site per month	2.9±3.22

Regarding healthcare utilization, 45 (26.47%) had visited a health facility in the past 30 days. Twenty-nine (64.44%) visited private clinics including "Bengali doctors" (popular quacks in the community), 12 (26.66%) visited a government facility, and four (8.89%) visited a pharmacy. The mean±SD number of visits to the TI site by the transgender women was 2.9±3.22 times per month.

Reasons for visiting a government facility were as follows (as reported): "My treatment is ongoing there" and "I was referred". Reasons for choosing pharmacy/private clinics were the following: "Easy and quick", "The doctor is from our community", and "The doctor understands us". The most reported problem in availing the healthcare service was non-availability of separate queues for transgender persons: "If we stand in the queue for men, we are harassed and humiliated, and in the women's queue also, they don't want us to stand with them". Some participants (24, 14.11%) reported that they faced problems while visiting the TI site. The reported problems were as follows: people misbehaving, eve-teasing, passing comments on their commute, and distance and time constraints (Table 3).

**Table 3:** Healthcare facility preferences and challenges faced by transgender women in accessing services

Variable	Reason/number (%)
Reasons for visiting a government facility	Ongoing treatment, referral from another facility
Reasons for choosing a private facility	Doctor from the "own" (transgender) community, better understanding by the provider
Reasons for choosing a pharmacy	Easy and quick access
Reported problem while visiting a facility	Non-availability of separate queues for transgender persons
Participants reporting problems while visiting the targeted intervention site (n=170)	24 (14.11%)
Problems faced while visiting the targeted intervention site	Misbehavior by people while travelling, eve-teasing, passing comments during commute, and distance and time constraints

**Figure 1:**
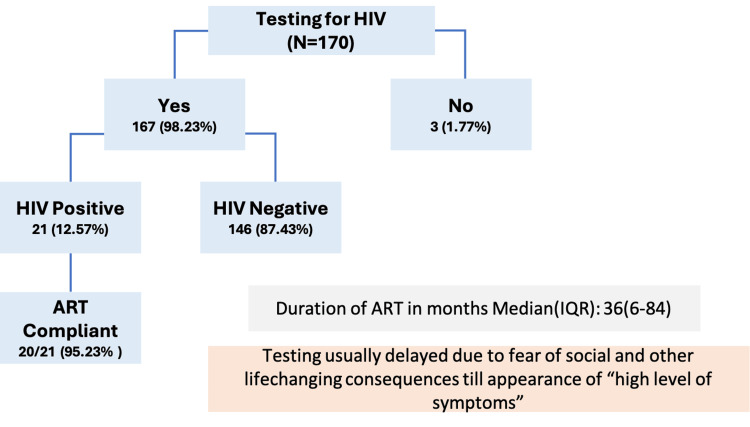
Service utilization: testing for HIV at the TI site HIV: human immunodeficiency virus; ART: antiretroviral therapy; TI: targeted intervention

Most participants, 167 (98.2%), underwent periodic HIV testing at the TI site, with 21 (12.5%) testing positive (Figure 1).

**Figure 2:**
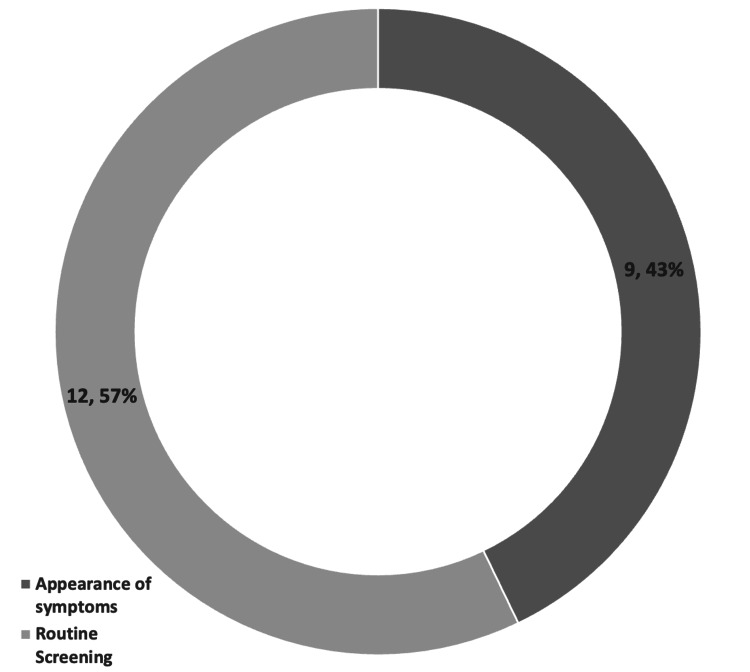
Service utilization: reasons for undergoing HIV testing HIV: human immunodeficiency virus

Many delayed the HIV testing due to fear of social consequences. Around 43% (9/21) got the screening test done only after the appearance of symptoms (Figure 2). 

Those on ART had treatment for a median duration of 36 months. Non-compliance with ART was found in one participant who had discontinued treatment for three years. The reported reason for the discontinuation of ART in the participant was an interruption in accessibility due to the COVID-19 pandemic. The mean (SD) distance of the ART center where treatment was being availed was 10.5 (6.36) km. Only a few (8, 38.1%) of the transgender PLHIV reported that their family/friend/partner/living community were aware of their HIV status. Most of the transgender PLHIV (15, 71.4%) said that they visit the ART center "alone always" for treatment and follow-up.

Factors that act as barriers to HIV services

Internalized Stigma

Internalized stigma often affected the effective utilization of available health services. "I did something wrong. I am a hijra now, and it does not seem nice to contact my family. They will not be happy to see me. The neighbors will laugh at them." "Sometimes I think getting abused may be right for me" (HIV-positive study participants describing that though she wants to meet her birth family, she will never be able to face them).

Health System and Lack of Trans-supportive Care

Many doctors lacked knowledge about transgender health needs. Study participants reported that healthcare providers' limited understanding of transgender issues often impacts their disease management. "Doctors ask us awkward questions" and "Doctors do not know about us; they do not know what organs we have and what we don't have" (transgender woman during FGD).

Queues in healthcare facilities often resulted in emotional distress and abuse. When standing in the male queue, they frequently faced verbal harassment, ridicule, and even physical intimidation from men. On the other hand, joining the female queue provoked derogatory comments from women. Due to the absence of transgender-specific restrooms even in private hospitals, inpatient admissions often became costly for participants. They frequently had to reserve rooms with attached toilets, which were more expensive.

Prevalent Prejudice and Myths About HIV Within the Transgender Community

It was often observed that the study participants would only discuss HIV in hushed tones, believing that speaking about it loudly could attract the disease to them and their community. HIV was often mentioned as "lambe taak" (prolonged duration). There were prevalent myths about how the infection (HIV) spreads. Often mentioned were "hugging", "having food together", "sharing a common kitchen", "sharing a common house", and "through clothes".

**Figure 3:**
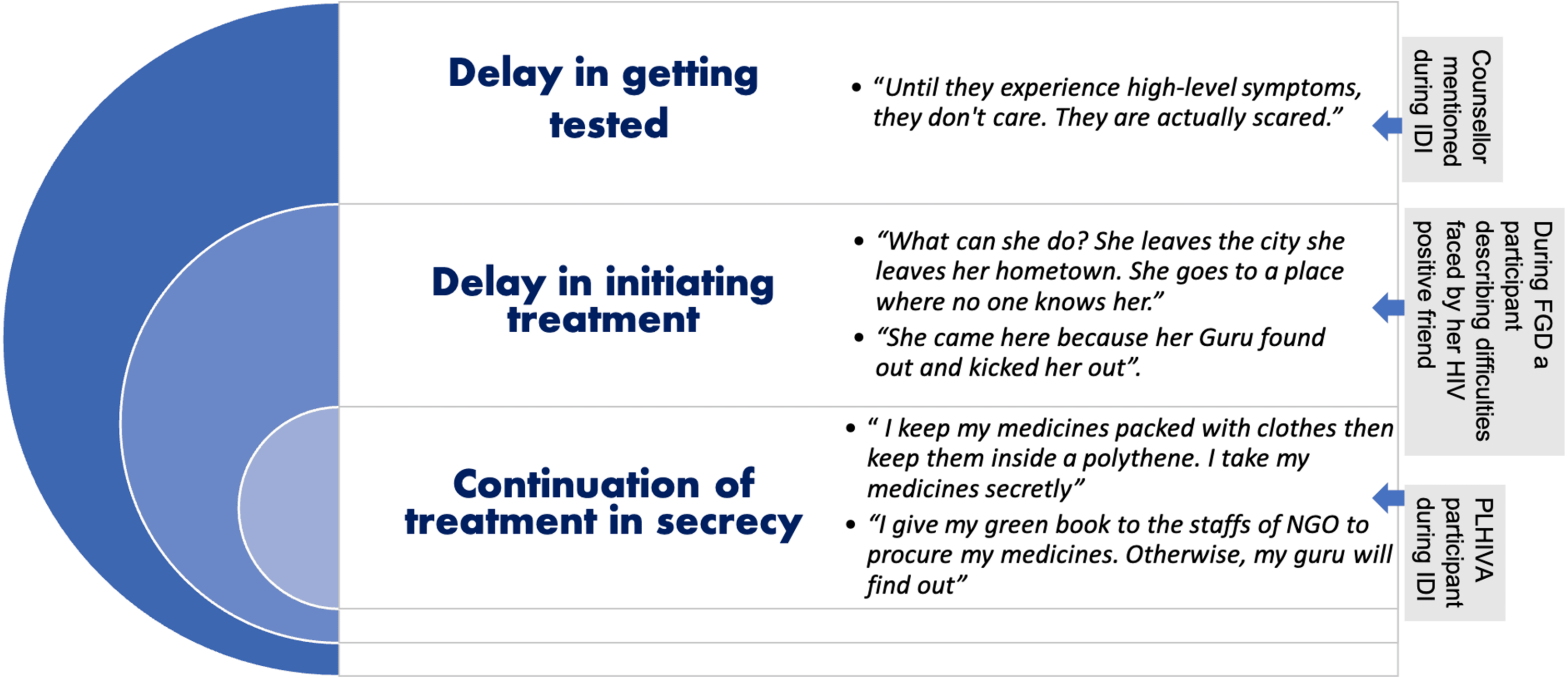
Peer rejection of a HIV-positive person in the transgender community: fear of adverse social consequences HIV: human immunodeficiency virus; FGD: focus group discussion; IDI: in-depth interview; NGO: non-governmental organization; PLHIVA: people living with HIV/AIDS Image created by the authors using PowerPoint (Microsoft Corporation, Redmond, Washington, United States)

Fear of adverse social consequences often resulted in delay in testing and delay in initiating treatment, and when initiated, they continued the treatment in secrecy (Figure 3).

Consequences of Being HIV Positive

An HIV-positive person is typically no longer accepted by the transgender community. "If someone finds out that a person is HIV positive, their utensils are separated; their bed is separated. And after 15 days, they are told that there is no room in their house, and they should find somewhere else to go. They call someone else and say, 'Don't let the woman who came to your house use the toilet, she has lambe taak (a name by which HIV infection is often referred to by the study participants; the literal meaning is prolonged duration)" (counsellor).

An HIV-positive individual is often deserted by their partner. "I had a boyfriend; we had been together for 12-13 years. After finding out I was HIV positive, I told him. And he was shocked. We stayed together for a few days, and then he left" (PLHIV participant describing her experience).

"If she showed her partner the green book, or the partner even glanced at the green book, he would immediately suspect that an HIV test was conducted, ART treatment is ongoing, and her partner will definitely leave her" (counsellor).

Fear of Being Seen Accessing HIV-Related Services

"Think about it, if someone sees her (in a government hospital), either a guru (community leader) or a chela (disciple of the guru), the whole community will come to know and will defame her. After that, they won't even accept whether she has the disease or not" (counsellor).

"Many transgender individuals wear masks when they go to the hospital, some wear burqas, and some wear veils to avoid being recognized" (laboratory technician).

Instead, they preferred to get their tests done at private facilities at "much higher costs" or call personnel from diagnostic laboratories or "path labs" to their homes.

Partner Notification and Follow-Up

Partner notification is difficult, a "challenging situation" as mentioned by the counsellor, as the infection is associated with stigma and health consequences for both the positive person and her partner. Partner follow-up is more difficult as usually the partners "just disappear" (counsellor reported during IDI).

Difficulty in Condom Negotiation: Unprotected Sex as Demanded by Clients

The majority of study participants identified "unsafe sex" as the main transmission route for HIV/AIDS. While they acknowledged the availability and accessibility of condoms and lubricants, there were also instances of unprotected sexual activities, often requested by clients of sex workers. "…my clients tell me to do it (sex work) without protection and offer an extra Rs 1000-2000" (transwoman narrating her experience).

Unprotected Sex Often Associated With Substance Abuse

"If you're unemployed and don't have a place to stay, you might turn to drinking, attend parties, and engage in casual sexual activities" (as quoted by a transwoman during FGD describing how one may have unprotected sex because of psychological disturbances despite knowing the associated risk). "It is very difficult to do sex work without drinking" (transwoman mentioning during interview).

The Linkage of a Transgender Person With an NGO

A medical officer mentioned: "I think the main barrier is to connect a TG to the NGOs who are working in this field as without the assistance of NGOs, it's extremely challenging for transgender individuals to sustain as they frequently lack family support and face discrimination".

Table 4 lists the factors that act as barriers to HIV services.

**Table 4:** Factors which worked as barriers for HIV services HIV: human immunodeficiency virus; NGO: non-governmental organization

Theme/category	Description/findings
Internalized stigma	Feelings of shame and self-blame were reported. This affected the seeking of healthcare services and connection with family
Health system and lack of trans-supportive care	Limited provider knowledge regarding transgender health needs, insensitive interactions, absence of transgender-friendly infrastructure, and being abused in gender-segregated queues
Prevalent prejudice and myths about HIV within the transgender community	Misconceptions regarding HIV transmission and fear associated with discussing HIV openly contributed to delayed testing, delayed treatment initiation, and secrecy around treatment
Consequences of being HIV positive	HIV-positive transgender individuals often experienced social isolation, exclusion from the transgender community, separation of personal belongings, housing insecurity, and abandonment by intimate partners
Fear of being seen accessing HIV-related services	Fear of disclosure, stigma, and community defamation affected the utilization of government HIV services. Testing from private providers or home-based sample collection was preferred despite higher costs
Partner notification and follow-up	Was challenging due to the associated stigma of partners becoming untraceable after diagnosis
Difficulty in condom negotiation	Financial incentives from clients and power imbalance during sex work often led to unprotected sexual practices despite awareness regarding HIV prevention
Unprotected sex associated with substance abuse	Alcohol and substance use, housing insecurity, and psychological distress contributed to inconsistent condom use
Non-availability or delayed NGO linkage and support	Non-availability or delayed availability of NGO services as they played a crucial role in supporting transgender individuals through healthcare linkage, social support, and assistance in overcoming discrimination and lack of family support

Facilitators for Availing HIV Services

Awareness About Symptoms and Route of Transmission

It was seen in the quantitative part of the study that all the study participants were aware of the common symptoms of HIV infection and mentioned unsafe sex and sharing of needles as routes of transmission.

Availability of Services at the TI Site

The study participants also mentioned the easy availability and accessibility of condoms and lubricants and health education and medical consultation from a doctor.

Enabling Social Environment at the TI Site

Another facilitating factor mentioned was the presence of other members of the transgender community. They have fewer medical but more social problems noted by the medical officer. "Discussion on grooming, dress design, weight loss, etc. are always helpful for me to visit" (transwoman mentioned during interaction). One participant shared, "Other people like us (transgender female) come here, and we can be like a woman without being judged or ridiculed".

Support From TI/NGO Staff During Social Conflicts

Support during interpersonal or social conflicts by the NGO staff played a role. It was reported, "When my neighbors were harassing me, Didi (elder sister, here with reference to the social worker) went and talked to them, now they don't bother me as they (neighbors) know I am not alone".

Representation From the Transgender Community

Having representatives from the transgender community improved trust and made the participants avail the services offered by the TI site. "Better results are achieved when people from the community talk to them" and "One of our counsellors and many staff here belong to the trans community" (laboratory technicians explaining that the representative of the transgender community in their organization helps them to build a connection).

Financial Assistance Schemes by the State Government

The financial assistance scheme for PLHIV provided by the Delhi State Government was frequently cited by transgender individuals living with HIV as one of the major sources of livelihood. "The money helps me manage my medicines, food, and rent. Without it, surviving would be very difficult" and "This scheme gives me security for daily expenses" (participants mentioned during IDI).

Table 5 presents the factors that act as facilitators for availing HIV services.

**Table 5:** Factors which worked as facilitators for availing HIV services HIV: human immunodeficiency virus; TI: targeted intervention; NGO: non-governmental organization; PLHIV: people living with HIV/AIDS

Theme/category	Description/findings
Awareness regarding HIV symptoms and transmission	All participants were aware of the common symptoms of HIV infection and identified unsafe sexual practices and sharing of needles as major routes of transmission
Availability of services at the TI site	Availability of HIV testing facilities, condoms, lubricants/jelly, health education sessions, and medical consultation encouraged visits to the TI site
Supportive social environment at the TI site	Presence of peers from the transgender community created a non-judgmental and safe space, encouraging participants to visit the TI site
Support from TI/NGO staff during social conflicts	NGO staff members provided support during interpersonal and community conflicts
Representation from the transgender community	Presence of counsellors and staff members from the transgender community improved trust
Financial assistance schemes by the state government	Financial assistance provided by the Delhi State Government for PLHIV served as a major source of livelihood for transgender individuals living with HIV

## Discussion

The findings of this study reveal a range of challenges faced by transgender women in accessing HIV-related services in the context of a broader sociocultural environment. Transgender women often lack familial and societal support. With the revelation of their transgender identity, they faced refusal or were charged higher rents for accommodation. Due to societal discrimination, they are often relegated to occupations commonly prevalent in the transgender community including sex work [12,13].

As reflected in participant narratives, internalized stigma, that is, the fear of being judged by family and society, discouraged them from reconnecting with their birth families or seeking timely healthcare services. This also reduces the likelihood of retention in HIV care as reported [14].

The healthcare system itself posed numerous challenges for transgender women. Participants frequently reported uncomfortable encounters with physicians, and this has been reported from other countries as well [15,16]. Additionally, the absence of trans-supportive facilities, such as separate queues and transgender-specific restrooms, created significant discomfort and forced them to reserve private rooms with attached bathrooms during inpatient stays, which were financially burdensome. Previous international and Indian studies have highlighted a significant lack of transgender-sensitive healthcare services [17,18]. These findings are consistent with the World Professional Association for Transgender Health (WPATH) Standards of Care Version 8, which emphasize the importance of gender-affirming healthcare environments, respectful communication, provider competency in transgender health, and reduction of stigma within healthcare settings [19]. In this context, the recent reform of the undergraduate medical education curriculum of the country by the National Medical Commission of India provides an excellent opportunity [20,21]. The National Medical Commission, the statutory regulatory body that oversees medical education in India, has also advised removing all content in the teaching curriculum that can be perceived as derogatory to this specific community [22].

The study revealed widespread misconceptions about HIV transmission within the transgender community. Myths about transmission through casual contact, such as hugging, sharing utensils, or using the same kitchen, were prevalent. These misconceptions fuelled fear and secrecy, preventing open discussions about HIV prevention and treatment. Terms like "lambe taak" were used to avoid directly referring to HIV, reinforcing stigma and misinformation.

The fear of social rejection was a recurring theme, leading to delayed testing, late initiation of treatment, and efforts to continue treatment in secrecy. Counsellors and participants shared stories of being abandoned by family, partners, and the larger community upon disclosure of their HIV status. Stigma and fear associated with HIV/AIDS, combined with social isolation and financial hardship, further discourage them from accessing HIV-related services. Research conducted in Nepal revealed high levels of healthcare stigma, discrimination, and societal prejudice, highlighting the challenging sociocultural landscape that transgender individuals usually go through [23].

Many transgender women avoided public health facilities out of fear of being recognized by members of their community. This fear of exposure often led them to wear masks, veils, or even burqas while seeking services or to opt for private diagnostic services at higher costs which further added to their financial burden.

Partner notification and follow-up proved to be complex and sensitive issues. We need a more specific program and data related to partner notification and service delivery in order to reduce the transmission of HIV [24].

Although condoms and lubricants were generally accessible as reported in another study from India [10], many participants reported engaging in unprotected sex at the insistence of clients who offered additional payment. Substance use often accompanied these encounters, further increasing the risk of unsafe sexual practices. Participants described how substance use helped them cope with psychological distress and made it easier to engage in sex work, despite knowing the associated risks. Interventions for transgender women in sex work should include comprehensive HIV education, mental health and substance use support, and condom negotiation training [25]. Access to pre-exposure prophylaxis and harm reduction programs will empower transgender women to prioritize safer sex and improve overall well-being [26].

The critical role of NGOs in supporting transgender individuals was highlighted by the medical officer. Participants frequently visited TI sites not only for medical services, including HIV testing and ART, but also for social support. These findings are corroborated by research emphasizing the importance of peer outreach workers and supportive healthcare providers in facilitating ART adherence and retention [27].

This study aims to understand the healthcare needs of transgender individuals from both the perspectives of beneficiaries and service providers. The results align with previous research, confirming existing challenges and gaps in the health system. The findings offer insights that can serve as a foundation for future studies. Additionally, they provide a useful resource for policymakers, healthcare providers, and advocates to design more inclusive policies and improve services that better meet the unique needs of transgender communities.

The study has certain limitations. Firstly, both the quantitative and qualitative components of the study were carried out using convenience-based approaches for selecting the study area and sampling procedure and determining the sample size. As a result, selection bias cannot be ruled out, and the study participants may not be representative of the larger population of transgender women. Still, our study likely shows the experiences of transgender women with similar traits who visit public health centers in Delhi. Secondly, the results may not be applicable to other regions of India due to cultural differences. Thirdly, the study was based largely on self-reported data, and the possibility of information bias cannot be excluded. Furthermore, social desirability bias may have influenced responses to sensitive questions related to HIV and healthcare access, and recall bias may have affected the accuracy of participants' recollection of past events and experiences. Additionally, while established qualitative rigor-enhancing procedures such as formal assessment of data saturation and independent coding by multiple researchers could have further strengthened the trustworthiness of the findings, these were beyond the scope and available resources of the present study. These methodological considerations should be taken into account while interpreting the study findings.

## Conclusions

This study demonstrates that access to HIV prevention and treatment services among transgender women is shaped by a complex interplay of social, economic, community, and health system factors. Persistent stigma and discrimination, coupled with financial insecurity and limited social support, continue to hinder timely access to and sustained engagement with HIV-related care. Systemic barriers, such as the absence of transgender-sensitive health services and facing discriminatory behavior while accessing services, create additional barriers to access.

This study underscores the urgent need for interventions to reduce stigma in society and strengthen healthcare providers' capacity to address the health needs of transgender individuals. This may be achieved through targeted training in transgender health and gender-affirming care, as well as greater integration of transgender health competencies into undergraduate and postgraduate medical curricula. Facilitation of economic independence through skill enhancement and employment opportunities may decrease the dependence on high-risk livelihoods like sex work and lower the risk of HIV transmission among transgender women. Further research involving broader and more diverse transgender populations is needed from developing countries like India.

## Appendices

**Table 6:** Semi-structured interview schedule HIV: human immunodeficiency virus; ART: antiretroviral therapy; TI: targeted intervention; NGO: non-governmental organization

Identification details		
Q. no.	Variable/question	Response options
1	Serial number	
2	Date of interview	___/___/___
3	Name/code of participant	
4	Age	
5	Address/area of residence	
6	Contact number	
7	Duration of stay in Delhi	
Sociodemographic profile		
8	Current living arrangement	Alone/Guru-chela household/Family/Partner/Other
9	Relationship status	Single/In a relationship/Married/Separated-Divorced/Widowed
10	Educational status: ___ years	
11	Occupation	Sex work/Badhai/Job/Business/Daily wage/Unemployed/Other
12	Monthly income	
13	Substance use	Tobacco/Alcohol/Ganja/Drugs/None/Other
Health problems and service utilization		
14	Current health problems	Yes/No
15	If yes, specify	
16	Visits to the TI facility	___/month
17	Visited a healthcare facility in the last month	Yes/No
18	Type of facility visited	Government hospital/Private clinic/Medicine shop/NGO-TI/Other
19	Reason for choosing the facility	
HIV and treatment information		
20	HIV testing done	Yes/No
21	HIV testing part of	Routine/Symptom-based request/Any other reason
22	Aware of HIV status	Yes/No
23	Duration since HIV diagnosis	
24	Duration on ART	
25	ART medication regularity	Regular/Sometimes/Stopped
26	Current ART center	
Knowledge: HIV/AIDS		
27	Signs/symptoms of HIV/AIDS	
28	Mode of spread	
29	Prevention methods	
Access to ART and healthcare services		
30	Difficulty coming to the ART/TI center	Yes/No
31	Difficulties faced	Transport/Financial/Distance/Stigma/Lack of support/Work issues/Other
32	Problems accessing healthcare services	Yes/No
33	If yes to Q. no. 33, problems at the facility	Waiting time/Stigma/Poor staff behavior/Lack of privacy/ID issues/Timing/Other
Facilitators for utilization		
34	What factors helped you in utilizing ART and healthcare services? (Use probes if required) (Record participant responses verbatim as far as possible)	(Probes: Accessibility, medicines, friendly staff, privacy, transgender-friendly care, guru-community support, family support, financial support, awareness)
Barriers to utilization		
35	What factors prevent or make it difficult for you to utilize healthcare or ART services? (Use probe if required) (Record participant responses verbatim as far as possible)	(Probes: Distance, waiting time, stigma, poor attitude, financial issues, disclosure fear, violence, work commitments, side effects)
Suggestions and remarks		
36	Suggestions for improvement	
37	Remarks/observation	
